# Extracellular vesicles isolated from dsRNA-sprayed barley plants exhibit no growth inhibition or gene silencing in *Fusarium graminearum*

**DOI:** 10.1186/s40694-022-00143-w

**Published:** 2022-07-14

**Authors:** Timo Schlemmer, Richard Lischka, Linus Wegner, Katrin Ehlers, Dagmar Biedenkopf, Aline Koch

**Affiliations:** 1 Centre for BioSystems, Land Use and Nutrition, Institute of Phytopathology, Justus Liebig University, Heinrich-Buff-Ring 26, 35392 Giessen, Germany; 2 Institute of Phytomedicine, University of Hohenheim, Otto-Sander-Strasse 5, 70599 Stuttgart, Germany; 3 Intitute of Botany, Justus Liebig University, Heinrich-Buff-Ring 38, 35292 Giessen, Germany

**Keywords:** Extracellular vesicles, Plant EVs, Barley, *Fusarium graminearum*, RNAi, dsRNA-based pesticides, Spray-induced gene silencing, RNAi-based plant protection, dsRNA, siRNA

## Abstract

Numerous reports have shown that incorporating a double-stranded RNA (dsRNA)-expressing transgene into plants or applying dsRNA by spraying it onto their leaves successfully protects them against invading pathogens exploiting the mechanism of RNA interference (RNAi). How dsRNAs or siRNAs are transferred between donor host cells and recipient fungal cells is largely unknown. It is speculated that plant extracellular vesicles (EVs) function as RNA shuttles between plants and their pathogens. Recently, we found that EVs isolated from host-induced gene silencing (HIGS) or spray-induced gene silencing (SIGS) plants contained dsRNA-derived siRNAs. In this study, we evaluated whether isolated EVs from dsRNA-sprayed barley (*Hordeum vulgare*) plants affected the growth of the phytopathogenic ascomycete *Fusarium graminearum*. Encouraged by our previous finding that dropping barley-derived EVs on *F. graminearum* cultures caused fungal stress phenotypes, we conducted an in vitro growth experiment in microtiter plates where we co-cultivated *F. graminearum* with plant EVs isolated from dsRNA-sprayed barley leaves. We observed that co-cultivation of *F. graminearum* macroconidia with barley EVs did not affect fungal growth. Furthermore, plant EVs containing SIGS-derived siRNA appeared not to affect *F. graminearum* growth and showed no gene silencing activity on *F. graminearum CYP51* genes. Based on our findings, we concluded that either the amount of SIGS-derived siRNA was insufficient to induce target gene silencing in *F. graminearum,* indicating that the role of EVs in SIGS is minor, or that *F. graminearum* uptake of plant EVs from liquid cultures was inefficient or impossible.

**Supplementary Information:**

The online version contains supplementary material available at 10.1186/s40694-022-00143-w.

## Background

Research on plant extracellular vesicles (EVs) is an emerging field that has undergone rapid progress in the last three years, with more than 260 studies published (PubMed). The published evidence that plant EVs and vesicle-like nanoparticles (VLNs) exhibit beneficial effects on human health [53, 60] encouraged scientists to isolate macro- and nanosized vesicles from diverse food sources, e.g., *Panax ginseng* [10], *Asparagus cochinchinensis* [63, 64], *Aloe vera* [24], garlic [38], bitter melon [61], grapefruit [50], strawberry [39], carrot [23] and honey [9]. This allowed them to study their anti-inflammatory, anticancer, antioxidative, and antisenescence properties. Notably, since EV-specific markers are not yet available for plant products such as fruits and vegetables, their extracellular origin needs to be verified. However, the strong bioactivity and biocompatibility of EVs and VLNs together with their efficient cellular internalization (e.g., penetration into glioma tissues by receptor-mediated transcytosis; [35] have raised the possibility of exploiting them as novel drug delivery vehicles [62].

Although plant EVs were first described in the apoplast in 1967 [18], it was almost half a century before they were separated from plant apoplastic fluids and then visualized with transmission electron microscopy (TEM) [42, 43, 45]. These pioneering works have laid the foundation for examining the role or contribution of EVs during plant-pathogen interactions [6, 8, 12, 32, 59]. These examples intensify the assumption that plant- or pathogen-derived EVs contribute bidirectionally to this highly specialized interspecies communication through the release of lipids, proteins, and small RNAs (sRNAs) that regulate and deregulate defensive and offensive responses [5, 51]. In particular, the identification of plant EV-derived sRNAs stimulated a debate about whether EVs function as shuttles in interspecies communication, directing plant antifungal defence responses [2, 7, 44, 47–49]. Conversely, fungal pathogens secrete sRNAs to dampen plant immunity [13, 29, 57, 58]. This sRNA-based crosstalk, also known as cross-species RNAi, was first described by Weiberg et al. [57], demonstrating that the fungal pathogen *Botrytis cinerea* produces sRNAs that mimic plant sRNAs and bind to *A. thaliana* AGO1 to antagonistically silence important plant immunity genes [57]. Similarly to the suggested plant EV-mediated sRNA transport, it is proposed that fungal sRNA delivery is also facilitated by EVs [31]. To prove this hypothesis, EVs isolated from different fungal pathogens, such as *Ustilago maydis* [30], *Zymoseptoria tritici* [20], *Fusarium oxysporum* [4, 16] and *F. graminearum* [17, 48], were established, which lay the foundation for further study of cross-species RNA transport in plant-fungus interactions in the near future.

In agriculture, RNAi technologies attract immense scientific and political interest as powerful substitutes for conventional chemical pesticides to reach the EU’s and UN’s [14, 55] sustainable development goals [52]. Currently, RNAi-based plant protection relies on two strategies that differ based on the origin of the dsRNA utilized. In the first strategy, endogenous dsRNA formation mediated by transgene expression is designated as host-induced gene silencing (HIGS). The second strategy is based on exogenous, foliar dsRNA application known as spray-induced gene silencing (SIGS). Notably, the principle of cross-species RNAi was biotechnologically used (HIGS) [36] before its naturally occurring equivalent was discovered [57].

We previously demonstrated that a transgene-derived CYP3RNA (a dsRNA designed to target *CYP51A*, *CYP51B* and *CYP51C* genes in *F. graminearum*), as well as foliar application of CYP3RNA, induced *CYP51* target gene silencing in *F. graminearum* [25, 27]. Remarkably, HIGS-or SIGS-mediated *F. graminearum CYP51* downregulation conferred strong *F. graminearum* disease resistance in *Arabidopsis thaliana* (HIGS) and *Hordeum vulgare* (HIGS and SIGS) [3, 21, 25–27] Despite many proof-of-concept studies demonstrating the efficacy of RNAi in pathogen and pest control (for review, see: Koch and Kogel [65–69] [32, 41], our mechanistic knowledge of HIGS and SIGS is still incomplete, although researchers hope to translate testing from the lab to the field soon [41]. Understanding the routes by which dsRNAs and siRNAs are delivered into fungal cells will be key to further improving cellular uptake and systemic distribution, and therefore increasing the stability and efficacy of exogenously applied dsRNA-based pesticides.

Studying the role or requirement of EVs in transferring HIGS- and SIGS-associated RNAs, we recently showed that EVs isolated from CYP3RNA-expressing *A. thaliana* plants contain CYP3RNA-derived siRNAs [47]. Notably, subsequent differential digestive treatments of EVs with RNase, protease, and a detergent revealed that the amount of intravesicular siRNA was low [47], more than 70% of the CYP3RNA-derived siRNAs were found to be extravesicular. EVs isolated from CYP3RNA-sprayed barley plants revealed CYP3RNA-derived siRNAs, too; however, their abundance was even lower compared with EVs isolated from HIGS *A. thaliana* plants [49]. This difference might be due to the various dsRNA origins in HIGS and SIGS approaches, whereby sprayed RNAs must be taken up by plant cells before being packed into plant EVs [28]. CYP3RNA uptake into plant cells and its systemic spread via the phloem have been previously reported, as well as its apoplastic transport in the xylem [3, 25]. However, since the amount of dsRNA-spray-derived siRNA in barley EVs was low, we asked whether EVs are required for the delivery and uptake of exogenously applied dsRNA to induce SIGS in *F. graminearum*.

To address this question, we assessed whether EVs isolated from SIGS plants can induce *F. graminearum CYP51* target gene silencing and fungal growth inhibition. For this, we performed in vitro treatments of *F. graminearum* with EVs isolated from CYP3RNA-sprayed barley plants. Remarkably, we found no effects on *F. graminearum* expression of *CYP51* or growth, further underlining the importance of clarifying whether EV-mediated sRNA transport is required during SIGS-barley–*F. graminearum* interaction.

## Results

**Fig. 1:**
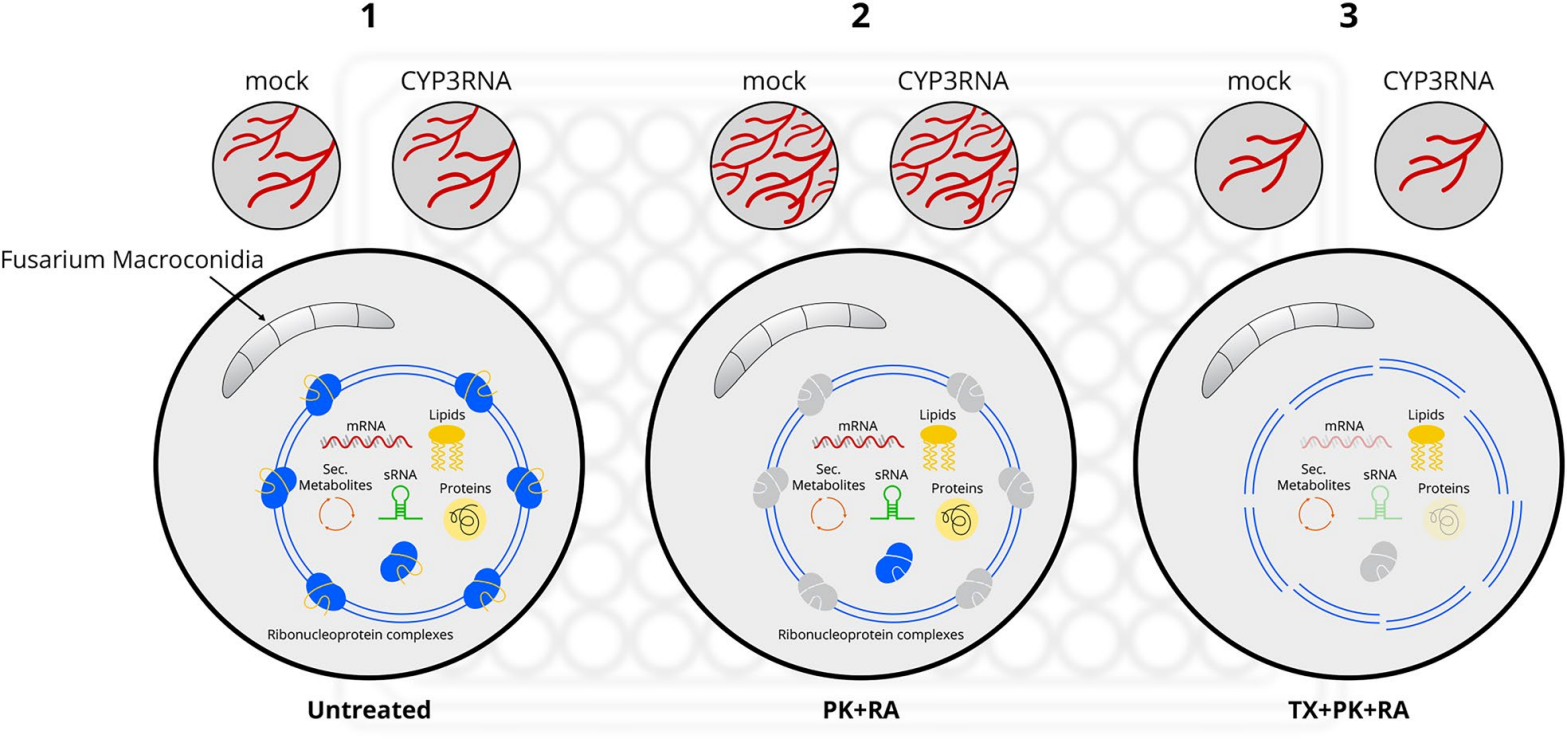
Schematic overview of the investigated EV treatments and their potential effect on EVs and their cargos. Fraction one (1) contained untreated EVs from mock or CYP3RNA-sprayed barley leaves and caused fungal growth. Fraction two (2) contained EVs treated with proteinase K (PK) and RNase A (RA) to degrade extravesicular ribonuclear complexes. Fraction three (3) contained EVs dissolved with triton X-100 (TX) and their cargo was degraded by PK and RA treatment

To test the possibility of plant EV uptake by *F. graminearum *in vitro, we isolated EVs from control [tris–EDTA (TE) buffer] and CYP3RNA-sprayed barley leaves using a protocol modified from those described by Rutter and Innes [45] and Schlemmer et al. [48]. In our recent studies, we observed that state-of-the-art EV purification from apoplastic fluids leads to impure EV isolates containing additional co-purified apoplastic substances [47]. This finding aligns with recent debates discussing the pitfalls of current plant EV research methods and the need for standardization, with different contamination risks reported for different plant EV separation and characterization methods [33, 40, 46]. To avoid such pitfalls that may lead to false conclusions, we performed a stringent digestive treatment of EV isolates to degrade extravesicular proteins and RNAs before in vitro treatment of *F. graminearum* with plant EVs. Each EV isolate was derived from 80 barley leaves and EVs were ultimately resuspended in 190 µl of phosphate-buffered saline (PBS). We reserved 40 µl for quality control measurements, TEM, and nanoparticle trafficking analysis (NTA). The remaining suspension was divided into three equal fractions (Fig. 1). To degrade extravesicular proteins, RNAs, and RNA–protein complexes, one fraction of EV isolates was treated with proteinase K and RNase A (PK + RA). In addition to PK + RA, the next fraction was treated with triton X-100 (TX + PK + RA), which is known to dissolve EVs [37, 45, 54] and denature extra- and intravesicular proteins, RNAs and RNA–protein complexes (Fig. 1). One fraction remained untreated to evaluate the effects of co-purified apoplastic fluid proteins or RNAs. Finally, EVs were co-inoculated with *F. graminearum* macroconidia and fungal growth was determined, after 20 h of pre-incubation, by optical density (OD) measurements every 20 min for a further 24 h.

**Fig. 2:**
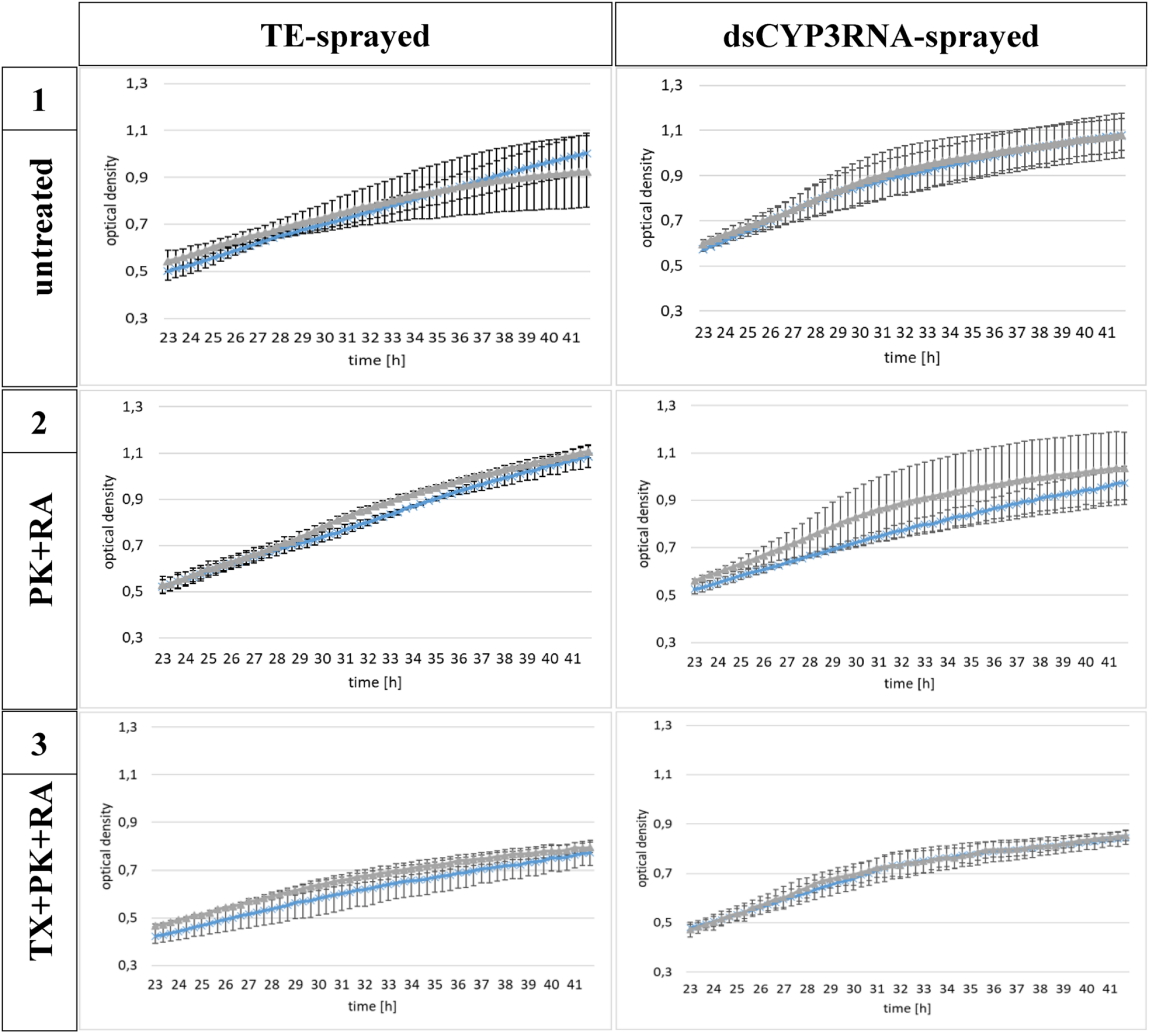
5 µl (light blue cross) and 10 µl (gray triangle) of purified EVs from the control (tris–EDTA) and CYP3RNA-sprayed barley leaves were added to *F. graminearum* liquid culture. Growth for cultures treated with EVs from all three fractions was determined by optical density measurements between 23 and 42 h post-inoculation (hpi)

To assess whether the effects depended on the investigated volumes, we used two different volumes of resuspended EV solution. We tested untreated EVs isolated from TE- or CYP3RNA-sprayed barley leaves and EVs treated with PK + RA and TX + PK + RA. We added 5 or 10 µl of each EV fraction to *F. graminearum* macroconidia*.* For each isolation, we investigated the same amount of barley leaves (80), which were previously sprayed either with TE or CYP3RNA; we then resuspended the EVs in the same volume of PBS before dividing them into the three fractions. Regardless of whether EVs were derived from CYP3RNA- or TE-sprayed barley leaves, or how EVs were treated after purification, no differences in *F. graminearum* growth were observed between treatment volumes (Fig. 2). At the beginning of the measurement period, 23 h post-inoculation (hpi), all samples showed an OD value of approximately 0.5. At 42 hpi, the OD for untreated and PK + RA-treated EVs had increased up to 0.9–1.1, while that for TX + PK + RA-treated EVs only rose to 0.7–0.9.

**Fig. 3:**
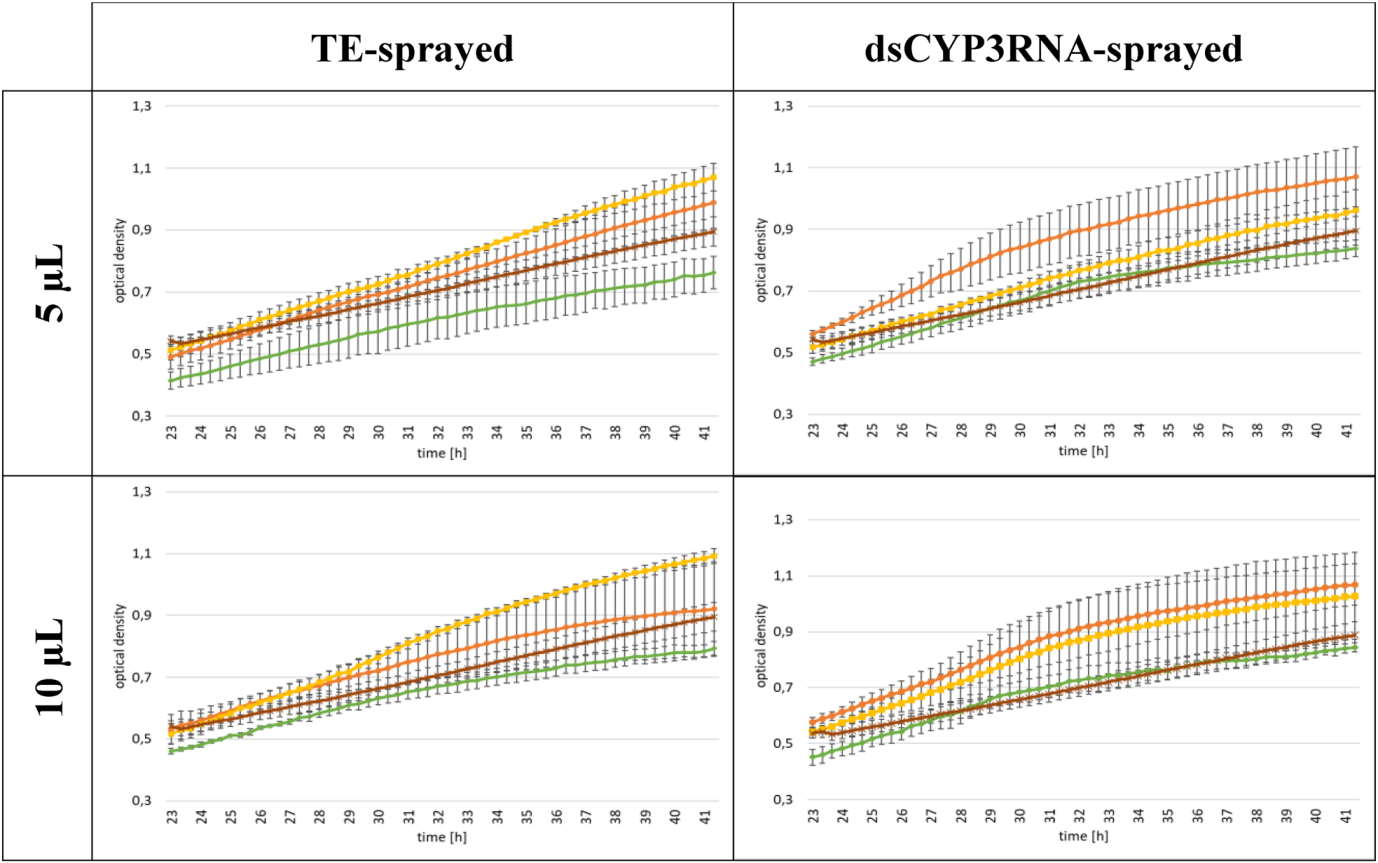
Purified barley EVs were treated with RNase A and proteinase K (yellow square) or Triton X-100, RNase A, and Proteinase K (green rhombus) after isolation and co-inoculated with *F. graminearum*. Additionally, untreated (orange circle) and EV-free PBS (brown cross) were co-inoculated as positive and negative controls

**Fig. 4:**
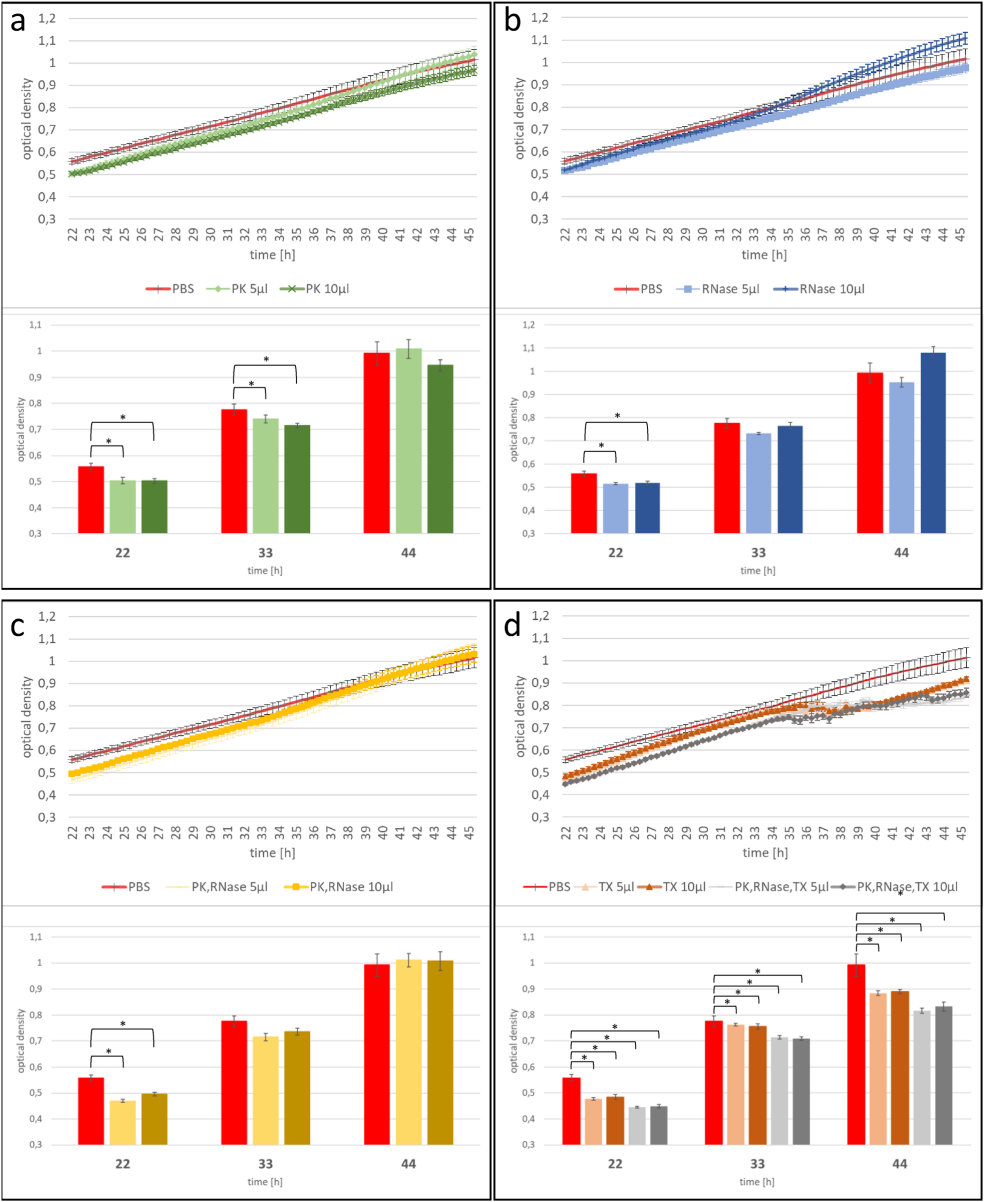
The effects of the investigated enzymes and detergent were evaluated by co-cultivating EVs without barley. 5 and 10 µl were added per enzyme, detergent, or combination. PBS (negative control, EV-free and enzyme- or detergent-free, red line) is shown as a reference. Optical density at selected timepoints [22, 33, and 44 h post-inoculation (hpi)] was mapped as a bar diagram and statistical analysis (two-tailed Student’s t-test) was performed with PBS-treated *F. graminearum* as reference. Asterisks indicate significant differences (*p*-value < 0.05)

**Fig. 5:**
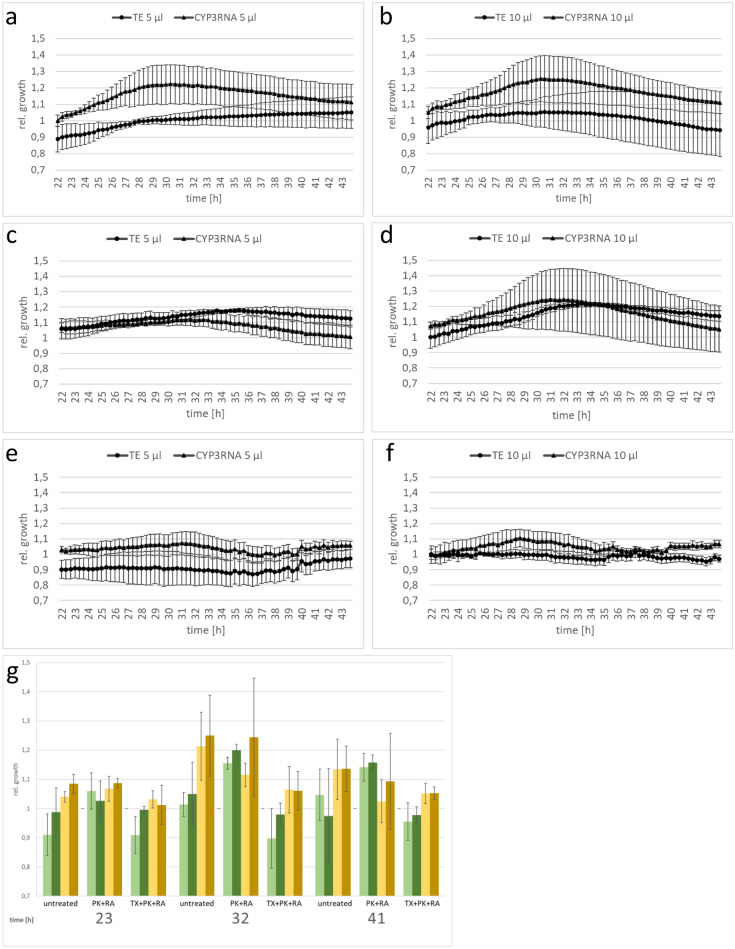
The relative fungal growth in the co-culture was calculated using the EV-free cultivation conditions with enzymes and detergent only as a baseline. Control (tris–EDTA): circle; CYP3RNA: triangle (**a**–**f**). Selected timepoints were chosen for statistical analysis. Differences between TE- or CYP3RNA-sprayed barley leaves were calculated for each investigated volume and EV pre-treatment using a two-tailed Student’s t-test (*p*-value < 0.05). 5 µl EVs of TE-sprayed barley leaves: light green; 5 µl EVs of CYP3RNA-sprayed barley leaves: dark green; 10 µl EVs of TE-sprayed barley leaves: light brown; 10 µl EVs of CYP3RNA-sprayed barley leaves: dark brown (g)

**Fig. 6:**
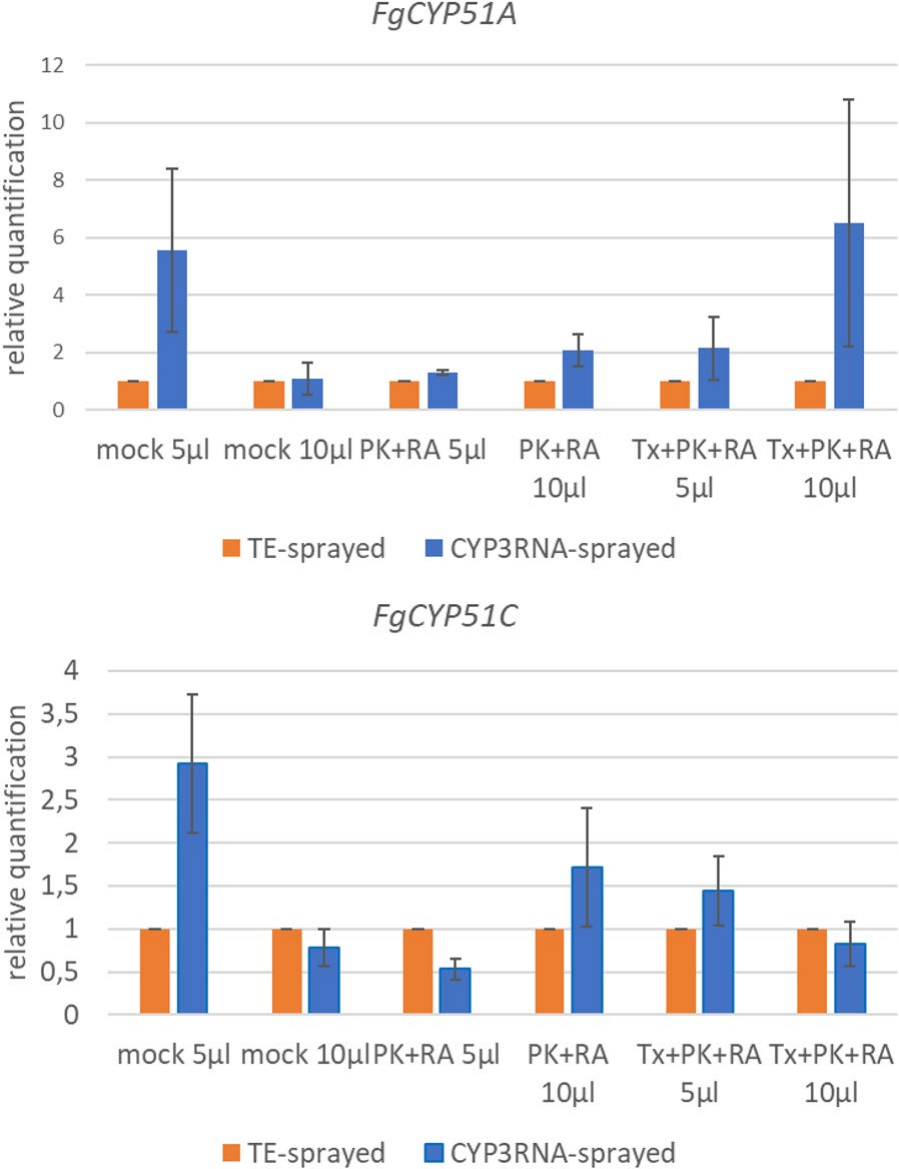
42 h post-inoculation (hpi) EV–*F. graminearum* co-cultures were harvested and technical triplicates were combined before RNA isolation. Transcriptional analyses were performed and *FgCYP51A* and *FgCYP51C* expression was calculated with the ΔΔCt method using the elongation factor 1 α as the reference gene. ΔΔCt values were calculated by referring to ΔCt values of samples which were derived from *F. graminearum* cultures where EVs were equally treated after isolation but derived from control-sprayed (non-RNA) leaves

As we observed no difference in the effect on fungal growth induced by different EV volumes, we next assessed the effect of EV treatments on *F. graminearum* growth. As a control, we used EV-free PBS, which was used as a buffer for EV resuspension after isolation. We compared the fungal growth over the measured time between the different EV samples. Focusing on *F. graminearum* growth with EVs from TE-sprayed barley leaves, we observed that PK + RA-treated EVs increased *F. graminearum* growth compared to PBS-treated *F. graminearum* cultures (Fig. 3). This was possibly triggered by simplified nutrient uptake via the degraded proteins and RNAs the enzymatic treatment created, or by the destruction of proteins that usually inhibit *F. graminearum* growth. However, we did not observe growth promotion when *F. graminearum* was fed with untreated EVs. The same observation was made when we focused on EVs from CYP3RNA-sprayed barley leaves, where no difference in fungal growth was observed after treatment of *F. graminearum* with different EV samples. Regardless of whether EVs originated from TE- or CYP3RNA-sprayed barley leaves, or whether 5 or 10 µl was applied, *F. graminearum* co-cultivated with TX + PK + RA-treated EVs was more inhibited than *F. graminearum* co-cultivated with PBS, untreated EVs, or PK + RA-treated EVs (Fig. 3). Therefore, we tested the detergent’s effect on *F. graminearum*. We mixed TX, PK, RA, PK + RA and TX + PK + RA with PBS, incubated them under the same conditions as the plant EVs and tested the mixtures in our growth assay. We observed no differences in the growth of *F. graminearum* treated with PK, RA, or a combination of both at the end of the growth assay (Fig. 4a–c). However, TX and TX + PK + RA reduced growth compared to the PBS control, indicating a clear effect of TX on fungal growth independent of plant EVs (Figs. 1; 4d). To avoid misinterpreting the effect of TX as that of CYP3RNA, we calculated the relative growth per EV treatment to compare the effects of TE- and CYP3RNA-sprayed EVs (Fig. 5). Remarkably, we found that CYP3RNA application did not inhibit growth, independently of how EVs were treated after isolation (Fig. 5). To verify this result and determine whether the unimpaired fungal growth could be explained by a lack of *F. graminearum CYP51* gene silencing, we isolated RNA from the *F. graminearum* cultures grown in microtiter plates and performed *F. graminearum CYP51* gene expression analysis. We found no downregulation of *CYP51* gene expression after co-cultivation with EVs isolated from CYP3RNA-sprayed barley leaves (Fig. 6). To test EV stability in PBS and the culture medium PDB, we added EVs resuspended in PBS to PBS or PDB in equal volumes. We froze samples in liquid nitrogen immediately after mixing and after 24 and 48 h of further incubation. The EVs were then analyzed using transmission electron microscopy (TEM) (Additional file 1: Fig. S1) and nanoparticle tracking analysis (NTA) (Additional file 2: Fig. S2). NTA measurements revealed low particle abundance, which was in line with the observations made by TEM. Therefore, we focused on close-up views of EVs and examined membrane integrity in the PBS and PDB medium at all three timepoints (Additional file 1: Fig. S1). Further, we harvested the cultured supernatant of *F. graminearum* after 24 h of pre-cultivation and added it to the EVs to test if they were degraded by secreted fungal enzymes such as lipases [56]. We measured particle concentration shortly after the fungal supernatant was administered to the EVs and 2 h post co-incubation. During this time, we saw no particle reduction, which suggests that the fungal culture supernatant did not cause any degradational processes in the EVs (Additional file 3: Fig. S3). Additionally, we repeated our fungal growth assay and investigated EVs from *A. thaliana* wildtype (wt) and double-stranded CYP3RNA-expressing transgenic plants. We observed that *A. thaliana* EVs had similar effects to those noted in barley EVs (Additional file 4: Fig. S4, Additional file 5: Fig. S5, Additional file 6: Fig. S6). No effects of the EV CYP3RNA cargo from HIGS plants were observed (Additional file 6: Fig. S6).

## Discussion

More than 50 studies demonstrate RNAi-based control of fungal pathogens with an average plant disease resistance of about 60% [28]. These studies reflect the enormous potential of RNAi technologies to meet the socio-political demand to halve the use of chemical pesticides by 2030 in Europe which has been approved by the European Commission in their farm-to-fork strategy in 2021 European Commission [70]. To meet this challenge, we must gain more mechanistic insights regarding the uptake and transport of exogenously applied dsRNAs to ensure their integrity and stability when used as dsRNA-based pesticides in the field. For example, proper RNA uptake and transport essentially serve as effective protection against degradation under difficult environmental conditions. Inside plants, exogenously-originating RNA may be further stabilized by the formation of RNA–protein complexes and/or EVs that can encapsulate RNAs, thus sheltering them from RNases or degradation in general during short- (cell-to-cell) or long-distance (systemic) movement. Given the assumption that EVs may participate in or facilitate the transfer of sRNAs during plant-pathogen interaction, the question is whether they are required in the transfer of HIGS- and SIGS-derived RNAs as well. We previously found that EVs isolated from HIGS-*A. thaliana* and SIGS-barley plants principally contain transgene- and dsRNA-spray-derived siRNAs [47, 49]. However, since the amount of HIGS and SIGS-related siRNA inside EVs was low, we assessed here whether these siRNAs could induce the silencing of *F. graminearum CYP51* genes and thus fungal growth inhibition, despite their low abundance.

To address these questions, we treated *F. graminearum* with EVs isolated from dsRNA-sprayed barley plants as well as transgenic HIGS-*A. thaliana* in vitro. The impurity of plant EV isolates raised concerns about the reliability of findings and their interpretation [33, 46]; we thus performed rigorous digestive treatments of EV isolates before *F. graminearum *in vitro testing. Encouraged by our previous finding that drop inoculation of barley EVs on *F. graminearum* cultures grown on solid agar plates caused an increase in purple pigmentation, indicative of the stress-induced premature formation of fruiting bodies [48], we expected to observe similar effects in liquid cultures. Interestingly, another recent study demonstrated the antifungal activity of EVs derived from root exudates of tomato plants against *F. oxysporum*, *B. cinerea* and *Alternaria alternata* [12], supporting the validity of in vitro EV–fungal spore interaction tests.

Surprisingly, we found that neither wild-type barley EVs nor EVs isolated from SIGS and HIGS led to inhibition of *F. graminearum* growth (Fig. 5; Additional file 6: Fig. S6). In addition, even different EV volumes (5 or 10 µl EV suspension) did not affect fungal growth (Fig. 2). In our previous successful experiments on solid agar plates, 40 µl of EV suspension derived from 80 barley leaves was drop-inoculated onto *F. graminearum*, suggesting that the volumes of 5 and 10 µl used in the present experiments might be too low. Given our previous finding that barley EVs led to stress-related discoloration of *F. graminearum* colonies [48] we assume that *F.* *graminearum* may be unable to take up EVs in vitro*.* A second possibility is that the amount of spray-derived siRNA in EVs is insufficient to induce fungal target gene silencing and the expected growth inhibition. To test the second possibility, we performed *F.* *graminearum CYP51* gene expression analysis on *F. graminearum* cultures after EV treatment, which was a more sensitive way to test CYP3RNA effects on *F. graminearum* than determining the OD of liquid fungal cultures. Underlining our results showing no growth inhibition in *F. graminearum*, we observed that EVs from CYP3RNA barley leaves did not show any gene-silencing activity (Fig. 6). However, this could still be explained by the inability of *F. graminearum* to take up plant EVs in vitro. Given this presumption, another study demonstrated sunflower-derived EV uptake by the ascomycete *Sclerotinia sclerotiorum* through reduced hyphae growth and spore germination [43], indicating that fungal uptake of plant EVs is possible in principle. In addition, recent studies indicated in vitro uptake of plant-derived (ginger, grapefruit, pineapple, and paprika) EVs and VLNs in human and rat cells [15, 22, 34], which is of great scientific interest due to their therapeutic potential in nanomedicine [11]. However, whether this holds for other fungal plant pathogens remains to be verified.

Notably, plant-derived EVs were shown to contain stress response-related proteins and lipids [8, 12, 32, 45, 47] and exhibit antifungal activity [12, 48]. It is therefore surprising that we did not observe any inhibitory effects of barley EVs on *F. graminearum*. This raises the question of whether EVs and their contents are stable in liquid media, and able to overcome the membrane or cellular barriers of *F. graminearum* and reach a defined threshold to activate the distinct RNAi machinery within its cells. To address concerns about EV stability in the resuspension buffer (PBS) and the cultivation media (PDB) we added barley EVs resuspended in PBS to PDB media or additional PBS (see detailed description in “Methods” section). At all three timepoints, which corresponded to the start of the experiment, the start of the OD measurements, and the end of the experiment, we observed EVs with intact membranes in both media (Additional file 1: Fig. S1). However, this assay does not provide any information on possible EV degradation by extracellular enzymes, e.g., fungal lipases secreted by *F. graminearum* Nguyen et al. [71]. Therefore, we tested the supernatant of 24-h-old *F. graminearum* cultures, which should have contained extracellular fungal enzymes together with barley EVs. There was no particle reduction measured after 2 h of co-incubation. Additionally, we previously isolated fungal EVs from *F. graminearum* which seemed to be resistant to lipases [48]. However, the lipid composition of plant and fungal EVs may be different.

Given our presumption that the low abundance of siRNA inside EVs may not be sufficient to induce a proper gene silencing response, the fundamental question about the relevance of EVs in transferring HIGS- and SIGS-associated RNAs remained. In addition to this, we already showed that more than 70% of HIGS-derived siRNAs were found to be extravesicular [47]. In support of this, the latest results from Roger Innes’ group have demonstrated that treatment of purified EVs with the protease trypsin and subsequent treatment with RNase A sufficiently eliminates RNA–protein complexes adhering to the outside of EVs, leading to the conclusion that extravesicular RNAs mediate HIGS rather than RNAs inside the EVs Zand Karimi et al. [72]. It is important to note that previous reports only rely on RNase treatment of purified plant EVs [7, 19]. Thus, missing the protease treatment may leave RNAs stabilized and protected from nuclease by RNA-binding proteins, which makes it impossible to distinguish between intra- and extravesicular RNAs and proteins. Given this assumption, it remains to be assessed why we observed no effect when fungal spores were treated with undigested EVs (where extravesicular RNA–protein complexes were intact) (Fig. 3, Additional file 5: Fig. S5). Further research is required to determine if this supports our presumption that *F. graminearum* is not able to take up EVs from in vitro liquid cultures, or if it is correct that even EV-adhering RNAs may not lead/contribute to HIGS. Together, these latest findings suggest that EVs may only play a minor or indirect role in the delivery and uptake of HIGS- and SIGS-associated RNAs. At least in the case of SIGS, this seems reasonable, because *F. graminearum* was shown to take up unprocessed dsRNA from the apoplast [25], and thus did not require the uptake of EVs for SIGS. It would therefore be interesting to elucidate the role or necessity of EVs in SIGS targeting of biotrophic fungal pathogens.

In summary, we found no inhibition of *F. graminearum* growth after treatment of in vitro cultures with SIGS- and HIGS-derived plant EVs. Subsequently, we found no *F. graminearum CYP51* target gene silencing, raising the question of whether *F.* *graminearum* is unable to take up EVs from a liquid culture or whether EV-contained and -adhering RNAs are insufficient to induce a proper gene silencing response in the species. However, further research is required to differentiate between improper EV uptake and the possibility that EVs may not play an important role in the translocation and uptake of RNAs in HIGS and SIGS.

## Conclusion

Mechanistic knowledge of RNA uptake and interspecies (plant–fungus) sRNA transfer is essential to the further development of RNAi technologies for plant protection. Here, we investigated the EV uptake ability of *F. graminearum* after in vitro treatment with SIGS-derived barley EVs. We found no growth inhibition or gene silencing in the species, indicating that either the fungus is unable to take up EVs from liquid cultures or the amount of RNA inside and/or outside the EVs is not sufficient to induce gene silencing of the target fungal genes. Our findings illustrate the importance of developing experimental readouts that allow the dependency of EV-mediated bidirectional sRNA transport for cross-species RNAi to be studied. In this context, studies have begun to identify and characterize plant and fungal EV content, as well as the importance of further developing EV isolation and purification protocols to improve reliability and avoid false interpretation of results. However, using EVs as natural blueprints may lead to the development of nanocarrier-based technologies that facilitate the efficient delivery of CRISPR/Cas9 components in the future [1]. In addition, fungal uptake of plant-derived EVs may offer potential routes to cure fungal diseases in humans, based on emerging evidence that plant-derived EVs exhibit great potential for human health applications [11].

## Methods

### EV isolation

*Arabidopsis thaliana* EVs were isolated from the apoplastic washing fluids of 90 plants per genotype. The apoplastic washes were harvested from the leaf rosettes, and then filtered through a 0.22 µm filter, centrifuged at 10,000×*g* and 100,000×*g* and resuspended in 190 µl PBS (8 mM NaH_2_PO_4_, 150 mM NaCl, 3 mM KCl and 2 mM KH_2_PO_4_; pH 7.4) [47]. The barley EV isolation protocol was adapted from the *A. thaliana* EV isolation described by Schlemmer et al. [49]. Each isolation included 80 barley leaves sprayed with tris–EDTA or CYP3RNA. Plant cultivation was performed in triplicate for both plant species and was followed by EV isolation, digestive treatment, and fungal co-cultivation assay.

### Differential EV treatments

Resuspended EVs were subdivided into three groups after isolation. The first group was untreated and served as a positive control (Table 1). The second group was treated with proteinase K and RNase A (PK + RA) and the third group with triton X-100, proteinase K, and RNase A (TX + PK + RA) (Table 1). All groups were incubated for 30 min at 37 °C and then added to *Fusarium graminearum* (*F. graminearum*) macroconidia.

**Table 1:** Components of the digestive EV treatments for eliminating intravesicular and apoplastic co-purified proteins and RNAs

Group	TE sprayed barley/wt *A. thaliana*			dsCYP3RNA sprayed barley/*A. thaliana* CYP3RNA		
	1	2	3	1	2	3
EV solution	50 µl	50 µl	50 µl	50 µl	50 µl	50 µl
RNase	–	1.2 µl	1.2 µl	–	1.2 µl	1.2 µl
PK	–	3 µl	3 µl	–	3 µl	3 µl
Triton X	–	–	5.8 µl	–	–	5.8 µl
PBS	10 µl	5.8 µl	–	10 µl	5.8 µl	–
Total	60 µl	60 µl	60 µl	60 µl	60 µl	60 µl

Investigated concentrations: Proteinase K (20 ng/µl) (Thermo Fisher Scientific); RNase A (20 ng/µl) (Thermo Fisher Scientific); 10% Triton X-100 (Sigma)

### Plant EV–*F. graminearum* co-culture assay

Plant EV–*F. graminearum* co-culture assays were performed in transparent 96-well plates with flat bottoms. PDB (potato dextrose broth, Formedium) was used as a carbon source. For macroconidia generation, *F. graminearum* strain IFA65 (IFA, Department of Agrobiotechnology, Tulln, Austria) was cultivated on synthetic nutrient-poor agar plates for 21 days at room temperature (RT) under constant illumination from one near-UV tube (Philips TLD 36 W/08) and one white-light tube (Phillips TLD 36 W/830HF). Macroconidia were washed off the plates with distilled water and filtered through sterile miracloth. 1 ml stock solutions were frozen in liquid nitrogen and stored at − 80 °C. All investigated stocks were derived from the same propagation event. One stock was thawed on ice per co-cultivation assay and macroconidia concentration (272,000 macroconidia/ml) was determined and adjusted to the investigated concentration by counting under a microscope in a Fuchs-Rosenthal counting chamber. Each well had 5440 macroconidia; 5 or 10 µl treated EV suspension and PBS were added (Table 2). 96-well plates were pre-incubated on the lab bench for 20 h before they were put into a plate reader (CLARIOstar, BMG Labtech) for another 24-h incubation at 25 °C with 60 rpm shaking. Optical density at 600 nm (OD600) was measured every 20 min in a 5 × 5 square pattern in each well. To exclude microbial contamination from EV isolates and prevent misinterpretation of optical density, one control (C) well contained no macroconidia (C1) (Table 3). Hygromycin was added to inhibit microbial growth and allow changes in optical density to be attributed to fungal growth (C2). C3 contained no PBS but rather an additional 0.5 × PDB. C4 contained no EVs. C3 and C4 were used to estimate the effect of the PBS on the optical density and growth behaviour of *F. graminearum*. As a reference for different EV treatments during the co-culture assay, the effects of EV treatment detergent were determined by incubating EV-free PBS with PK + RA (C5), TX + PK + RA (C6), PK (C7), RA (C8) and TX (C9) (Table 4). PBS was added to compensate for volume differences resulting from differences in the added volume of EV suspension. The co-cultivation was then performed according to the plant EV–*F. graminearum* cultivation method described in Table 5. Each experiment was performed in three wells and the means were taken for further analysis.

**Table 2:** Well composition for microtiter well co-cultivation of differentially treated plant EVs with *F. graminearum*

Group	1 (µl)	1 (µl)	2 (µl)	2 (µl)	3 (µl)	3 (µl)
Investigated vol	5	10	5	10	5	10
*F. graminearum*	20	20	20	20	20	20
0.5 PDB	125	125	125	125	125	125
PBS	10	5	10	5	10	5
Total	160	160	160	160	160	160

**Table 3:** Overview of tested controls and well composition

Controls	C1 (µl)	C2 (µl)	C3 (µl)	C4 (µl)
*F. graminearum*		20	20	20
0.5 PDB	160	125	140	125
PBS				15
Hygromycin		15		
Total	160	160	160	160

**Table 4:** Components of the digestive EV treatments used to measure the effects of treatment reagents on fungal growth

Controls	C5 (µl)	C6 (µl)	C7 (µl)	C8 (µl)	C9 (µl)
PBS	55.8	50	57	58.8	54.2
RNase	1.2	1.2		1.2	
PK	3	3	3		
Triton X		5.8			5.8
Total	60	60	60	60	60

Investigated concentrations: Proteinase K (20 ng/µl) (Thermo Fisher Scientific); RNase A (20 ng/µl) (Thermo Fisher Scientific); 10% Triton X-100 (Sigma)

**Table 5:** Well composition for microtiter well co-cultivation of EV-free detergent reagents to estimate treatment-dependent effects

Controls	C5 (µl)	C5 (µl)	C6 (µl)	C6 (µl)	C7 (µl)	C7 (µl)	C8 (µl)	C8 (µl)	C9 (µl)	C9 (µl)
Invest. vol	5	10	5	10	5	10	5	10	5	10
*F. graminearum*	20	20	20	20	20	20	20	20	20	20
0.5 PDB	125	125	125	125	125	125	125	125	125	125
PBS	10	5	10	5	10	5	10	5	10	5
Total	160	160	160	160	160	160	160	160	160	160

### *F. graminearum CYP51* gene silencing analysis

After 44 h of incubation, the cultures were transferred into new tubes for RNA extraction. The three technical replicates in the microtiter plate were merged to increase RNA outcome. 1 ml of GENEzol™ (geneaid) was added and extraction was performed according to the manufacturer’s instructions. cDNA synthesis was performed using QuantiTect ReverseTranscription kit (Qiagen). SYBER Green JumpStart Taq ReadyMix (Sigma-Aldrich) was used for qRT-PCR analysis of *F. graminearum CYP51A* and *CYP51C* genes as previously described [25, 27] (for primer sequences see [27]; Supplemental Table S2). Transcript levels of *CYP51* genes were determined via the 2^−ΔΔCt^ method by normalizing the amount of target transcript to the amount of translation elongation factor 1α. ΔCt values were calculated from three technical replicates. 2^−ΔΔCt^ values were calculated using three biological replicates.

### EV stability assay

PBS resuspended barley EVs were diluted 1:1 with PBS or PDB. The suspension was then carefully mixed by pipetting up and down several times and subdivided equally into three tubes. One tube of EVs mixed with PBS and one mixed with PDB were immediately frozen in liquid nitrogen. One tube per medium was incubated at 25 °C for 24 h and one for 48 h. Afterwards, samples were frozen in liquid nitrogen and stored at − 80 °C until nanoparticle tracking analysis (NTA) or transmission electron microscopy (TEM) were performed. For NTA measurements, samples were diluted at 1:20 with PBS and 200 µl were loaded into a Nanosight NS300 (Malvern Panalytical). Five measurements were performed at 25 °C and concentration prediction and size and statistical analyses were performed by the NTA 3.2 Dev Build 3.2.16 software. For TEM, copper formvar-coated 300-mesh electron microscopy grids were glow discharged before sample application for 40 s. Subsequently, 5 µl of each sample were applied onto its own grid. Samples were dabbed using Whatman filter paper and grids were washed three times in 50 µl of 2% uranyl acetate and once with distilled water. Excess staining or fixing solutions, buffers, and water were removed using Whatman paper between each step. Finally, the grids were air-dried. Preparations were inspected at 120 kV under zero-loss conditions (ZEISS EM912a/b) and images were recorded at slight underfocus using a cooled 2 × 2 k slow-scan CCD camera (SharpEye/TRS) and the iTEM software package (Olympus-SIS). At least ten meshes per grid were analyzed to avoid grid-to-grid variations.

To test if degradational processes of EVs are dependent on fungal exudates or extracellular enzymes, *F. graminearum* macroconidia were cultivated in PDB medium for 24 h. Fungal cells were depleted by centrifugation with 16,000×*g* at 4 °C for 10 min. 10 µl of fungal supernatant was added to 10 µl of barley EVs. Concentrations were determined by NTA after mixing fungal supernatant and barley EVs or after 2 h of incubation at 25 °C. Therefore, 180 µl of PBS was added for NTA measurements.

## Supplementary Information

**Additional file 1: Figure S1.** EVs were mixed with PBS and PDB and quick frozen at the beginning [0 h (h)], 24 h and 48 h after incubation at 25 °C. After fixing onto formvar-layered cupper meshes, samples were visualized with TEM. EVs were highlighted with red arrows.

**Additional file 2: Figure S2.** EVs were mixed with PBS and PDB and quick frozen at the beginning [0 h (h)], 24 h and 48 h after incubation at 25 °C. Samples were thawed on ice and diluted with PBS (1:20). The final volume of 200 µl was then loaded into the Nanosight NS300 (Malvern Panalytical) and five measurements were performed at room temperature. Mean values are arranged as a black line, standard deviation is given as the red plot.

**Additional file 3: Figure S3.** EVs were mixed with supernatant of 24-h-old *F. graminearum* culture and incubated at 25 °C. Particle concentration was determined by NTA measurements.

**Additional file 4: Figure S4.** 5 µl (light blue cross) and 10 µl (gray triangle) of purified EVs from control (wt) and CYP3RNA-expressing *A. thaliana* plants were added to *F. graminearum* liquid culture. Growth was determined by optical density measurements between 23 and 42 h post-inoculation (hpi) for cultures treated with EVs out of all three fractions.

**Additional file 5: Figure S5.** Purified *A. thaliana* EVs were treated with RNase A and proteinase K (yellow square) or Triton X-100, RNase A, and Proteinase K (green rhombus) after isolation and co-inoculated with *F. graminearum*. Additionally, untreated (orange circle) and EV-free PBS (brown cross) were co-inoculated as positive and negative controls.

**Additional file 6: Figure S6.** The relative fungal growth in the co-culture was calculated using the EV-free cultivation conditions with enzymes and detergent only as a baseline. Control (wt): circle; CYP3RNA-expressing plants: triangle (a-f). Selected timepoints were chosen for statistical analysis. Differences between wt or CYP3RNA-expressing *A. thaliana* plants were calculated for each investigated volume and EV pre-treatment using a two-tailed Student’s t-test (*p*-value < 0.05). 5 µl EVs of wt plants: light green; 5 µl EVs of CYP3RNA-expressing *A. thaliana* plants: dark green; 10 µl EVs of wt plants: light brown; 10 µl EVs of CYP3RNA-expressing *A. thaliana* plants: dark brown (g).

## Data Availability

All relevant data is contained within the article. The original contributions presented in the study are included in the article material, further inquiries can be directed to the corresponding author.
