# Supplementary figures and images for: Extracellular vesicles isolated from dsRNA-sprayed barley plants exhibit no growth inhibition or gene silencing in Fusarium graminearum
Source: Fungal Biol Biotechnol. 2022 Jul 14;9:14. doi: 10.1186/s40694-022-00143-w (PMC9284790; doi:10.1186/s40694-022-00143-w)

PBS

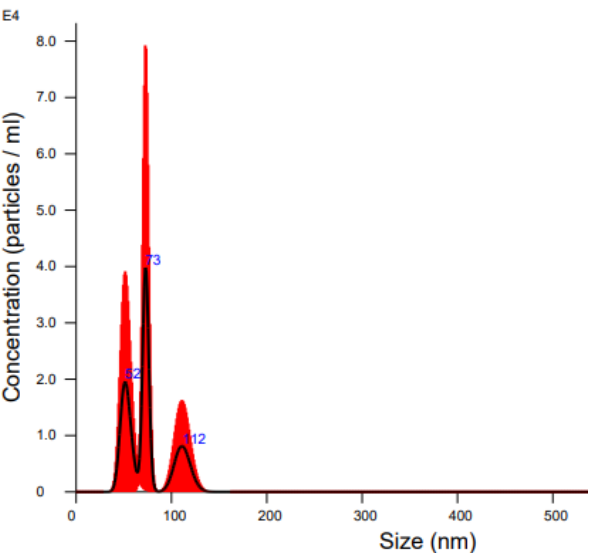

0 h

PDB

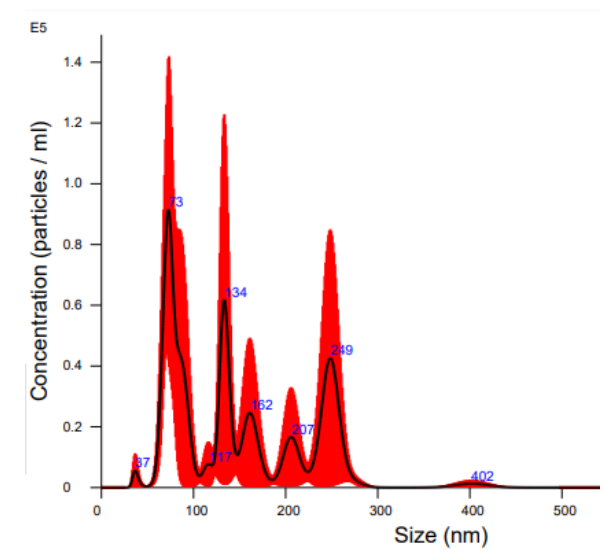

24 h

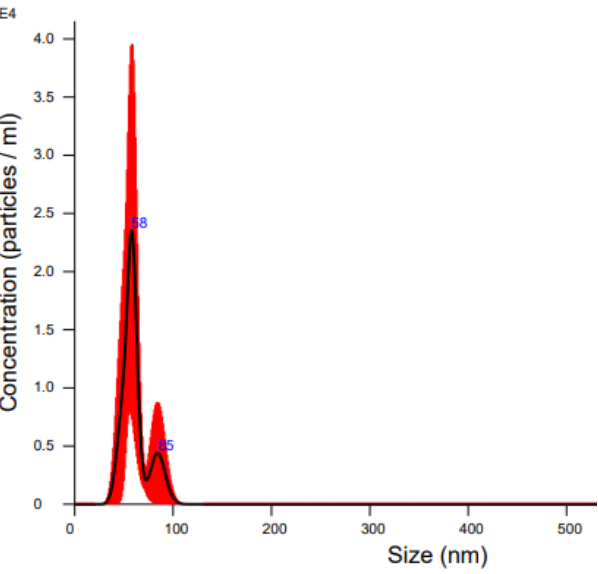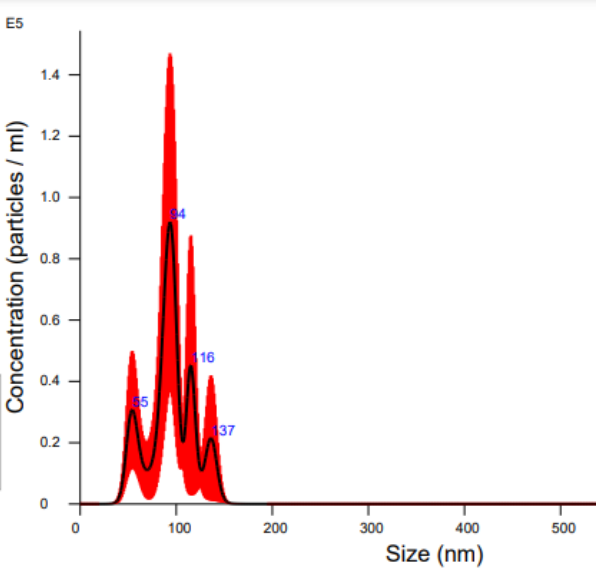

48 h

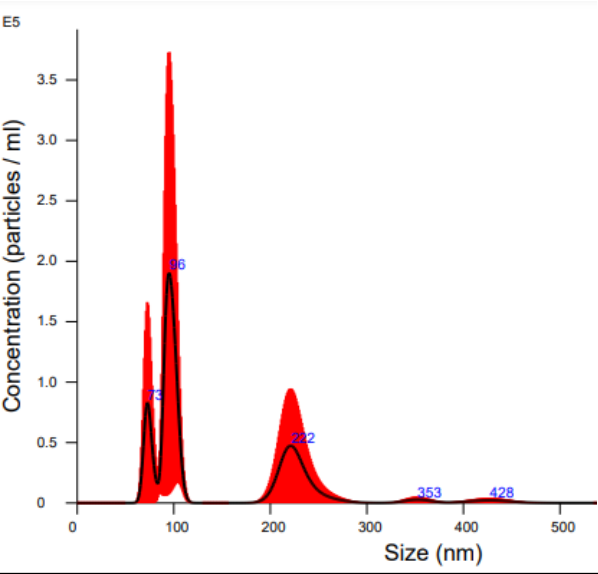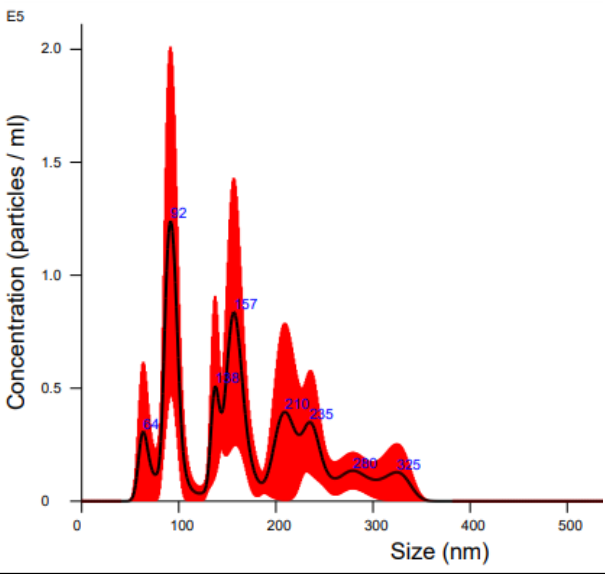

Supplement: Supplementary file 2 — Additional file 2: Figure S2. EVs were mixed with PBS and PDB and quick frozen at the beginning [0 h (h)], 24 h and 48 h after incubation at 25 °C. Samples were thawed on ice and diluted with PBS (1:20). The final volume of 200 µl was then loaded into the Nanosight NS300 (Malvern Panalytical) and five measurements were performed at room temperature. Mean values are arranged as a black line, standard deviation is given as the red plot. [file 40694_2022_143_MOESM2_ESM.pdf]

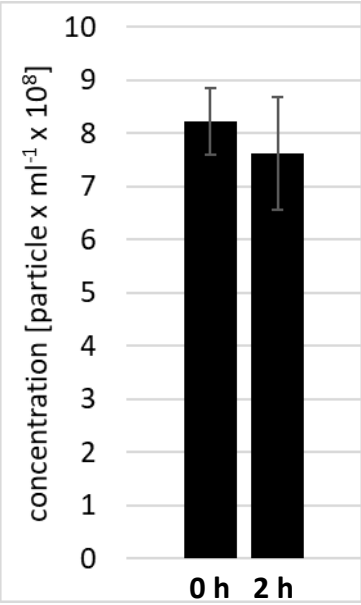

Supplement: Supplementary file 3 — Additional file 3: Figure S3. EVs were mixed with supernatant of 24-h-old F. graminearum culture and incubated at 25 °C. Particle concentration was determined by NTA measurements. [file 40694_2022_143_MOESM3_ESM.pdf]

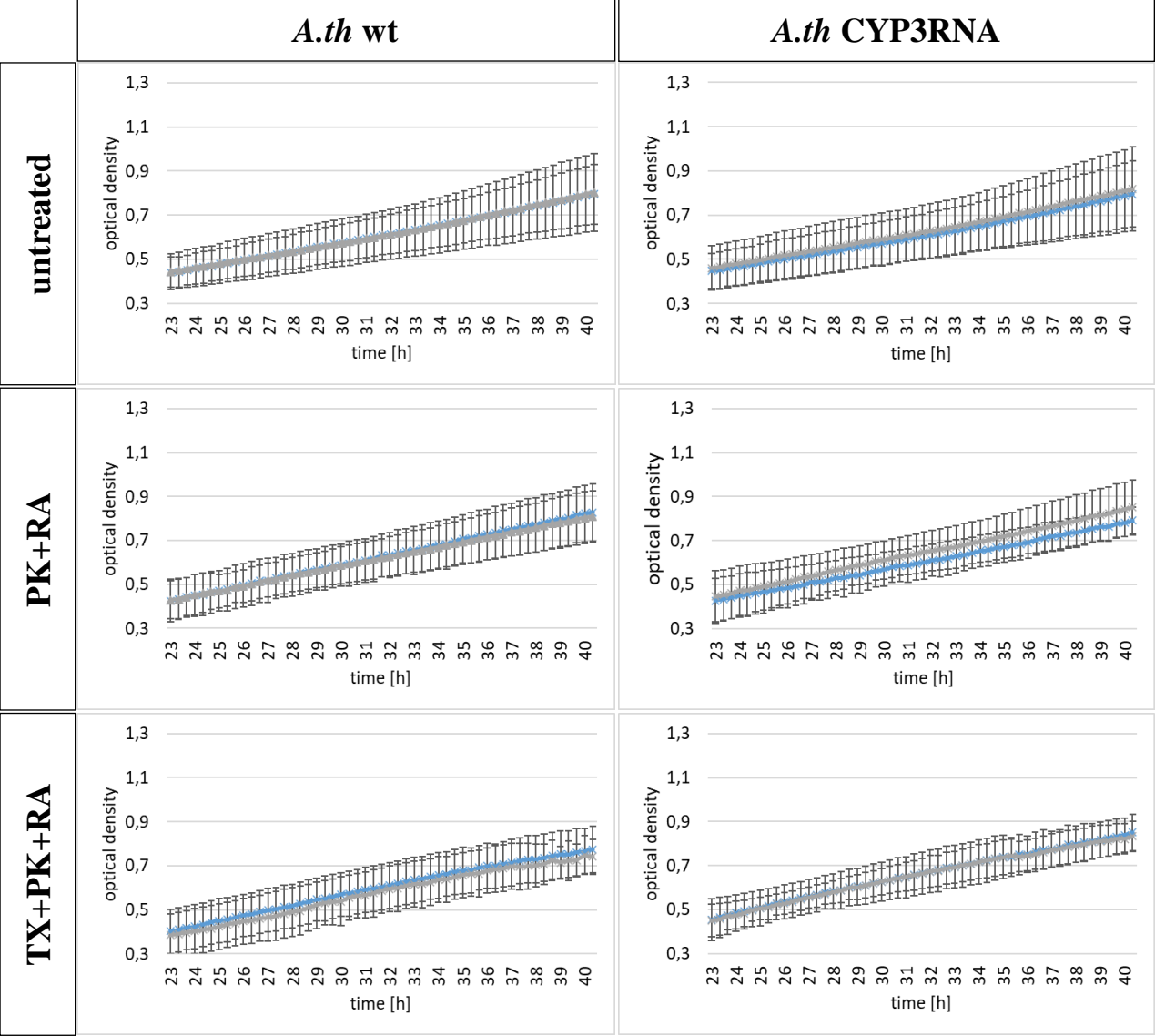

Supplement: Supplementary file 4 — Additional file 4: Figure S4. 5 µl (light blue cross) and 10 µl (gray triangle) of purified EVs from control (wt) and CYP3RNA-expressing A. thaliana plants were added to F. graminearum liquid culture. Growth was determined by optical density measurements between 23 and 42 h post-inoculation (hpi) for cultures treated with EVs out of all three fractions. [file 40694_2022_143_MOESM4_ESM.pdf]

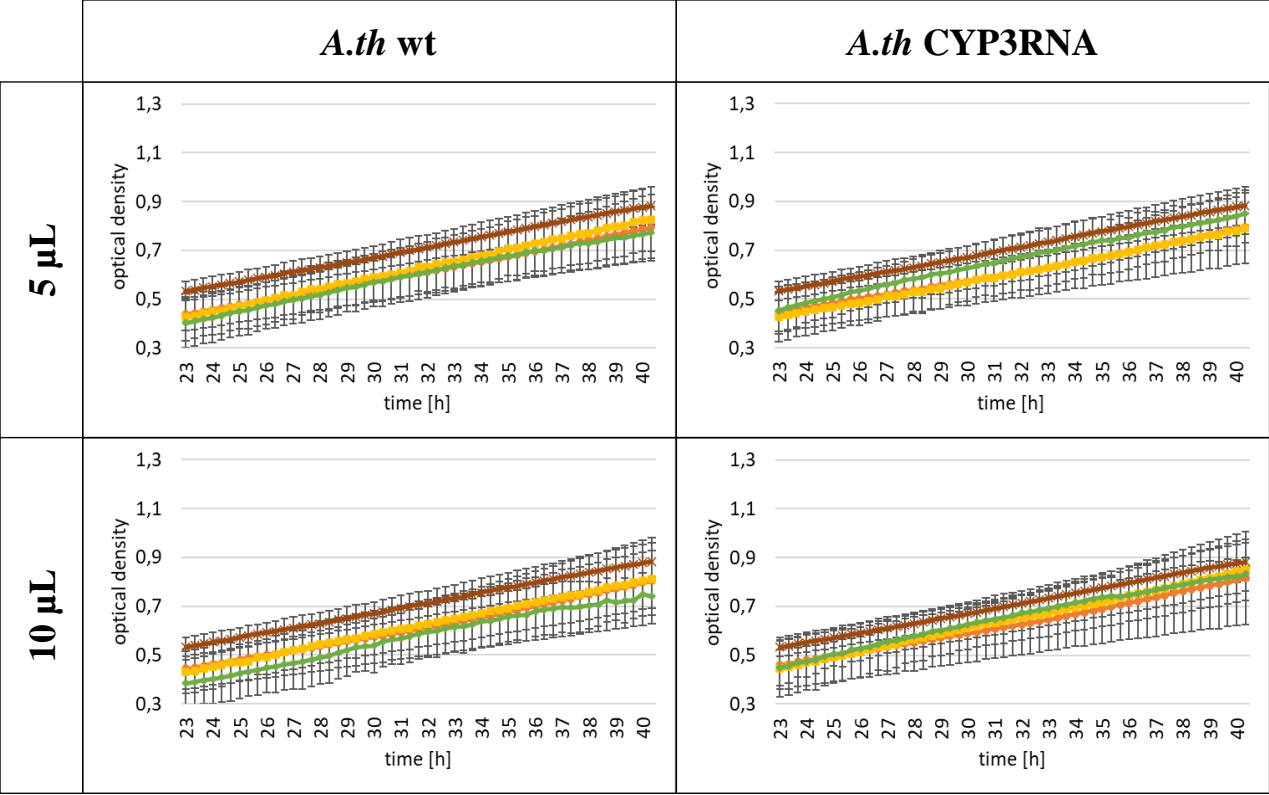

Supplement: Supplementary file 5 — Additional file 5: Figure S5. Purified A. thaliana EVs were treated with RNase A and proteinase K (yellow square) or Triton X-100, RNase A, and Proteinase K (green rhombus) after isolation and co-inoculated with F. graminearum. Additionally, untreated (orange circle) and EV-free PBS (brown cross) were co-inoculated as positive and negative controls. [file 40694_2022_143_MOESM5_ESM.pdf]

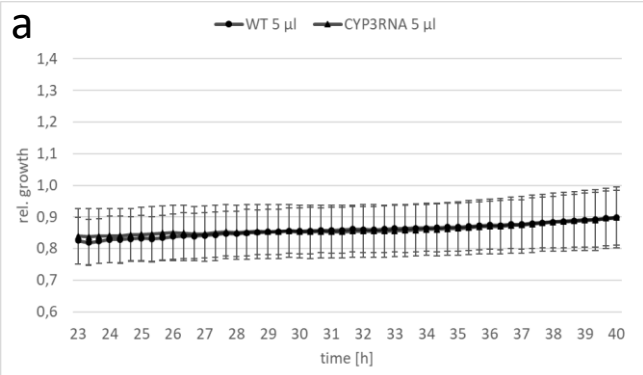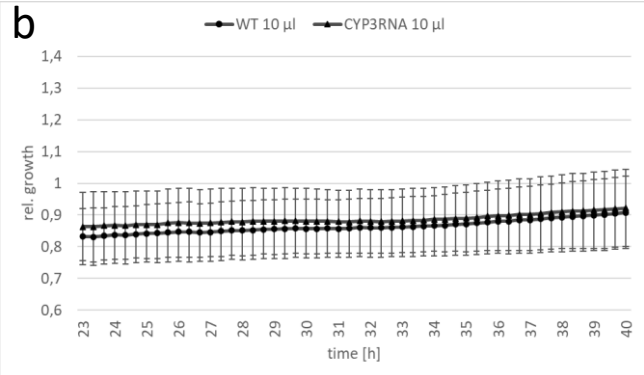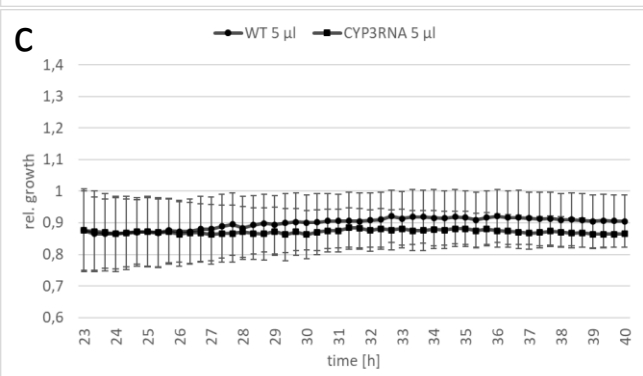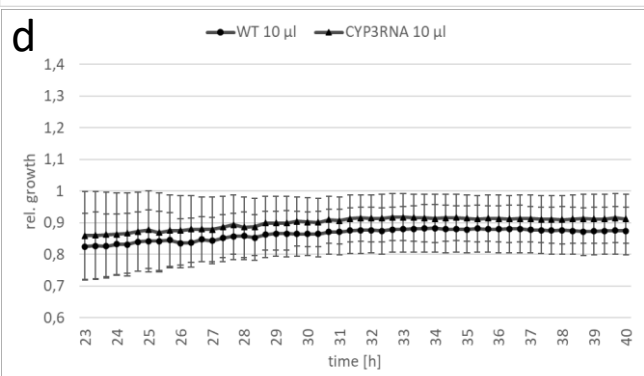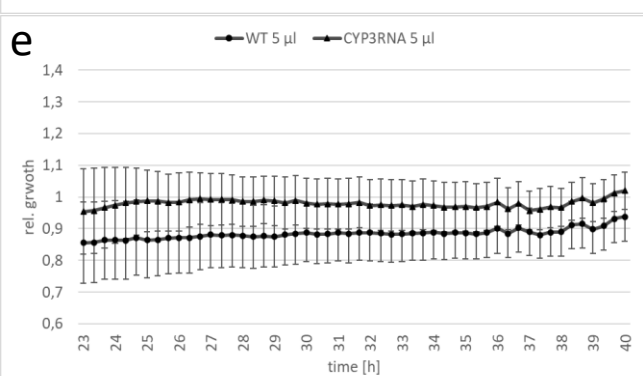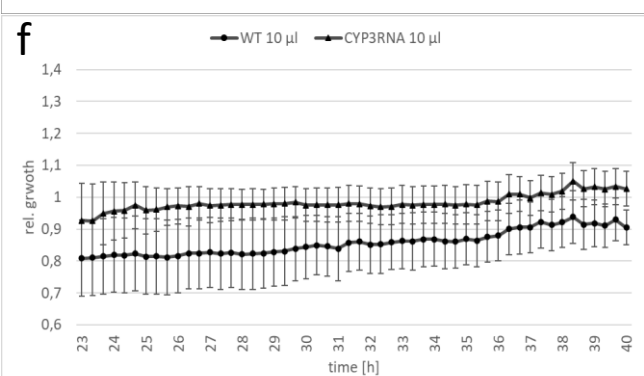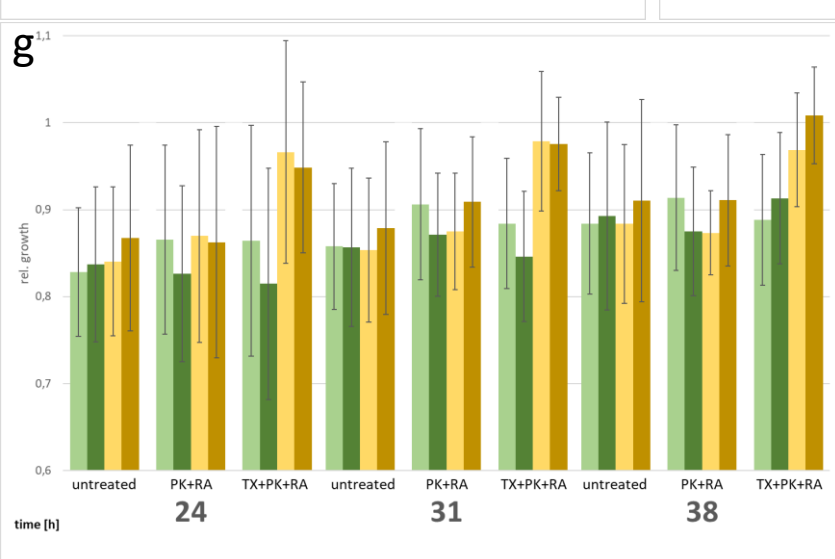

Supplement: Supplementary file 6 — Additional file 6: Figure S6. The relative fungal growth in the co-culture was calculated using the EV-free cultivation conditions with enzymes and detergent only as a baseline. Control (wt): circle; CYP3RNA-expressing plants: triangle (a-f). Selected timepoints were chosen for statistical analysis. Differences between wt or CYP3RNA-expressing A. thaliana plants were calculated for each investigated volume and EV pre-treatment using a two-tailed Student’s t-test (p-value < 0.05). 5 µl EVs of wt plants: light green; 5 µl EVs of CYP3RNA-expressing A. thaliana plants: dark green; 10 µl EVs of wt plants: light brown; 10 µl EVs of CYP3RNA-expressing A. thaliana plants: dark brown (g). [file 40694_2022_143_MOESM6_ESM.pdf]
